# sLithospermic acid etched ZIF-8 nanoparticles delays osteoarthritis progression by inhibiting inflammatory signaling pathways and rescuing mitochondrial damage

**DOI:** 10.1016/j.mtbio.2025.101589

**Published:** 2025-02-20

**Authors:** Yu Zhang, Qiqi Lou, Hao Lian, Ran Yang, Ruolin Cui, Leyang Wang, Bitao Ma, Lingli Hou, Lilun Jin, Weiran Teng

**Affiliations:** a Department of Traditional Chinese Medicine, School of Medicine, Xinhua Hospital, Afffliated to Shanghai Jiao Tong University, Shanghai, 200092, China; b Shanghai Institute of Precision Medicine, Shanghai Ninth People's Hospital, Shanghai Jiao Tong University School of Medicine, Shanghai, 200125, China

**Keywords:** Osteoarthritis, Synovitis, Lithospermic acid, Drug delivery, NF-κB pathway

## Abstract

Osteoarthritis (OA) is the most common chronic inflammatory joint disease. Improving the joint inflammatory microenvironment is expected to promote early intervention and delay the progression of OA. However, effective strategies for inhibiting OA-related joint inflammation are still lacking. Lithospermic acid (LA), a polycyclic phenol carboxylic acid extracted from salvia miltiorrhiza, has strong anti-inflammatory and antioxidant effects. However, its role in the treatment of OA and the underlying mechanisms are unclear. To improve the bioavailability of LA, an LA synergistic protects etched zeolitic imidazolate framework (ZIF)-8 nanoparticles (LA@ZIF-8) was designed and developed for targeted delivery to modulate the inflammatory microenvironment in OA. This study confirmed that LA@ZIF-8 inhibits the pro-inflammatory phenotype of RAW264.7 macrophages through the NF-ĸB signaling pathway, effectively alleviates mitochondrial dysfunction, and delays articular cartilage degeneration caused by the joint inflammatory microenvironment mediated by synoval macrophages. In summary, LA@ZIF-8 delays the progression of OA by inhibiting synovial macrophage-mediated inflammatory responses, highlighting its clinical application potential.

## Graphical abstract

**Figure:**
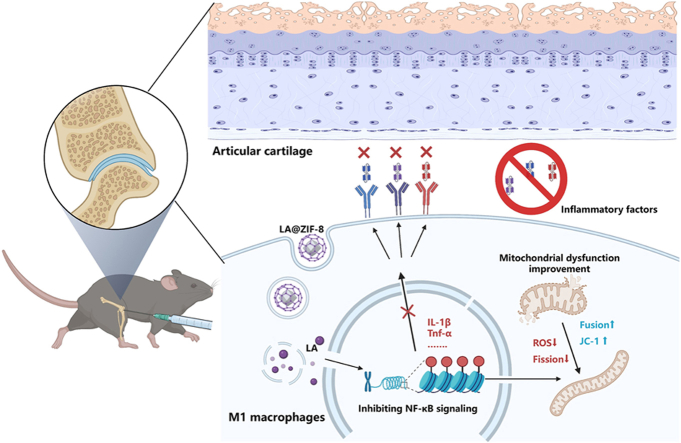

## 1 Introduction

Osteoarthritis (OA) is a low-grade inflammatory disease affecting the entire joint. The key pathological changes include articular cartilage injury, subchondral osteosclerosis, and synovitis [1,2]. There is growing evidence that inflammatory responses in the synovium play a key role in the development and progression of OA, leading to joint swelling and pain. OA, the most common joint disease, places a significant economic burden on individuals and society [3]. Current treatments for OA mainly involve local injections of glucocorticoids and oral non-steroidal anti-inflammatory drugs (NSAIDs), which only improve symptoms, such as joint pain, but do not delay the progression of the disease [4]. Therefore, there is a critical necessity to develop effective therapeutic drugs that can delay OA progression.

The joint microenvironment is crucial for the maintenance of cartilage homeostasis. Synovitis and cartilage degeneration are the two key pathological features of OA, with synovitis typically preceding cartilage degeneration [5]. OA synovitis primarily manifests as the differentiation of synovial macrophages into the M1 pro-inflammatory phenotype, which expresses various inflammatory mediators and induces oxidative stress. Mechanistic studies have found that M1 pro-inflammatory phenotypes is regulated by the mechanistic target of rapamycin, nuclear factor-kappa B (NF-ĸB), and PI3K/Akt signaling pathways, among others [6]. The activation of OA synovial macrophages produces various pro-inflammatory cytokines, such as interleukin (IL)-1β, IL-6, and tumor necrosis factor (TNF). These cytokines interact with chondrocytes through a paracrine pathway, causing chondrocyte inflammation and matrix degradation, leading to chondrocyte apoptosis and, by this means, exacerbating OA [7,8]. Furthermore, components such as chondrocyte extracellular matrix (ECM) degradation products continue to stimulate macrophage activation and exacerbate synovial inflammation, leading to cartilage damage [9]. Therefore, early inhibition of synovial inflammation and reduction of M1 polarization in synovial macrophages are suitable intervention targets for the treatment of OA. Inflammation induces oxidative stress, leading to mitochondrial damage and reactive oxygen species (ROS) accumulation [[10], [11], [12]]. Simultaneously, dysfunctional mitochondria release various mitochondrial components and metabolites into the cytoplasm, further driving the inflammatory response and promoting the secretion of IL-1β, IL-18, and other inflammatory factors [13,14]. Mitochondria are dynamic organelles that constantly fuse or divide, a process known as mitochondrial dynamics [15]. The process of fusion affects mitochondrial function, allowing mitochondria to form a network that favors oxidative phosphorylation (OXPHOS) and helps the cell resist the inflammatory environment [[16], [17], [18]]. Mitochondria that divide excessively into rings and fragments tend to produce high levels of ROS [19]. Thus, it is speculated that mitochondrial dysfunction in OA synovial macrophages causes more macrophages to undergo M1 polarization by inducing inflammation.

An increasing number of studies have confirmed that Chinese herbal medicines are effective in treating OA. Lithospermic acid (LA) is a polycyclic phenolic carboxylic acid derived from *S. miltiorrhiza*. It has strong anti-inflammatory [20], antioxidant [21], and antiviral effects [22], and has demonstrated therapeutic effects in alleviating nephrotic syndrome [23], mitigating acute myocardial ischemic injury [24], and improving liver fibrosis [25]. Studies have shown that LA effectively inhibits lipopolysaccharide (LPS)-induced macrophage inflammation [20]. However, The therapeutic efficacy of the traditional Chinese medicine monomer LA in vivo is limited due to its poor stability and difficulty in crossing the cell membrane barrier for absorption. Nano-drug delivery systems offer significant advantages over traditional treatment methods, including higher drug loading rates, greatly improved drug availability, and enhanced therapeutic effects [26]. Zeolitic imidazolate framework (ZIF)-8 is widely used as the basic framework for nanomedical drug delivery systems due to its easy preparation, good biocompatibility, and high stability [27]. Phenolic nanotechnology is an emerging field in biomedical applications, providing a simple and versatile method to assemble nanostructures, with unique properties and high flexibility. Natural polyphenolic compounds, such as tannic acid, are rich in phenolic hydroxyl, hydroxyl, benzene rings, and double bonds, and exhibit excellent chelating ability to metal ions [28]. Metal phenol complexes with unique properties and functions can be constructed by forming stable coordination bonds with metal ions. Phenolic acids are important adsorbents of heavy metal ions, and the organic complexes formed by their complexation with metal ions can effectively reduce their biological toxicity. When polyphenols and ZIF-8 are mixed in an aqueous solution, the polyphenols releases free protons, these free protons can be used to etch the structure of ZIF-8 [29]. In addition, the attached phenolic acid or polyphenolic acid molecules block the pores of the metal-organic framework (MOF) due to their large molecular size, thus protecting the outer part of the MOF from further etching. The metal-phenolic networks (MPNs) produced by polyphenol-etched ZIF-8 have nanoscale shells, contain multiple functional elements, and have reduced transmembrane resistance and biotoxicity [30].

We therefore hypothesised that LA can improve the joint inflammatory microenvironment by inhibiting macrophage inflammation, thereby treating OA cartilage injury. The LA@ZIF-8 nanoparticle was designed using ZIF-8 as a template and LA as an etching agent. We aimed to evaluate its biosafety in vivo and in vitro, screen and compare the inhibitory effect of the LA monomer and nanoparticles at appropriate concentrations on macrophage inflammation, and evaluate its effect on improving the OA inflammatory microenvironment and cartilage injury through co-culture with chondrocytes and intra-articular injection into the knee joint of OA mice. Transcriptome sequencing revealed the underlying mechanism of LA targeting macrophage inflammation treatment, offering valuable insights for the clinical development of innovative OA therapies and novel drug delivery systems. Continued research and development in this area has the potential to significantly advance the field of phenolic nanotechnology as well as cell therapy, providing new solutions for the treatment of various inflammatory diseases and conditions.

## 2 Methods

### 2.1 Reagent and instrument

LA was purchased from MedChemExpress (USA). LPS was purchased from Solarbio (Beijing, China). Dulbecco's Modified Eagle Medium (DMEM) (21068028) and DMEM-F12(11320082) were purchased from Thermo Fisher Scientific Inc(USA). Primary antibodies against TNF-α (17590-1-AP), IL-1β (16806-1-AP), iNOS (22226-1-AP), COX-2 (66351-1-PBS), Gapdh (10494-1-AP), Col2a1(28459-1-AP), Mmp13 (18165-1-AP), Mfn1 (13798-1-AP), Opa1 (27733-1-AP), Fis1 (10956-1-AP), Drp1 (12957-1-AP) were purchased from Proteintech (Wuhan, China), Sox9 (A19710) were purchased from Abclone (Wuhan, China), and phospho-P65 (3033S), P65 (8242S), IKKβ (8943), Phospho-IKKβ (2697) were purchased from Cell Signaling Technology(USA). Cell media, penicillin/streptomycin (P/S), 0.25 % trypsin-EDTA, and fetal bovine serum (FBS) were purchased from Gibco (Grand Island, USA). Primary antibodies Anti-ROS Assay Kit (S0033S), Enhanced Mitochondrial Membrane Assay Potential Kit (C2003S), Mito-Tracker Green (C1048), Fluorine 488-labeled Goat Anti-Rabbit IgG (A0423), Goat Anti-Lamouse (A0423), fluorine 555-labeled Donkey Anti-Rabbit IgG (A0453), fluorine 555-labeled Donkey Anti-Mouse IgG (A0460), Protease and phosphatase inhibitor cocktail (P1045) for General Purpose purchased from Beyotime Biotechnology (Shanghai, China). Insulin-transferrin-selenium (ITS) was from Gibco (51500056). TRIZol™ Plus RNA Purification Kit (12183555CN) was purchased from Thermo Fisher Scientific (USA). Hieff® qPCR SYBR Green Master Mix was purchased from Yeasen Biotechnology (Shanghai, China). Deionized water used in all experiments was prepared using a Milli-Q water purification system (Millipore, Boston, MA, USA).

### 2.2 Fabrication of ZIF-8 and LA@ZIF-8 nanoparticles

The development of nanometric ZIF-8 and LA@ZIF-8 particles was carried out in a mixed solvent environment with H_2_O and methanol. Before synthesis, 150 mg (mg) of zinc nitrate hexahydrate (Zn(NO_3_) _2_·6H_2_O) were dissolved in 5 mL (mL) of deionized water, while 165 mg of 2-methylimidazole were uniformly dispersed in 5 mL of methanol, Ultrasonic assisted dissolution. Lithospermic acid was carefully dissolved in methanol to obtain a drug concentration of 60 mg per milliliter (60 mg/mL). At the beginning of the synthesis, the Zn(NO_3_) _2_·6H_2_O solution (5 mL) was stirred vigorously at a speed of 500 revolutions per minute (rpm). Next, the 2-methylimidazole solution (5 mL) was carefully poured into the Zn(NO_3_) _2_ · 6H_2_O solution with vigorous stirring, and then 4 mL of methanol was added. After a 2-h reaction period at room temperature, ZIF-8 nanoparticles were collected by centrifugation, during which they underwent three thorough washes with methanol to remove undigested reagents. Mirroring the procedure for ZIF-8, 5 mL of 2-methylimidazole solution were gradually incorporated into Zn (NO_3_) _2_·6H_2_O stock solution (5 mL), then join 2 mL lithospermic acid solution, stirred at 500 rpm/min, then 4 mL of methanol was added. The stirred mixture was then allowed to react for 20 min at room temperature. The nanoscopic LA@ZIF-8 particles were then isolated by centrifugation at 12,000 rpm for 10 min, subjected to three exhaustive methanol washes to remove the unpackaged lithospermic acid. After vacuum lyophilization at −80 °C, nanoscale ZIF-8 and LA@ZIF-8 particles were prepared for further characterization efforts.

Preparation of ZIF-8 nanoparticles supported with fluorescein GFP or Rhodamine B (RhoB): 50 μg of GFP or Rhodamine B (RhoB) is added to 2-methylimidazole solution, and the subsequent synthesis steps are described above to synthesize nanoparticles with GFP or RhoB fluorescence.

### 2.3 Comprehensive characterization of LA@ZIF-8 nanoparticles

The characterization of ZIF-8 and LA@ZIF-8 nanoparticles was conducted through TEM (JEOL, Tokyo, Japan). The structure of the ZIF-8 and LA@ZIF-8 were monitored using X-ray diffractometer (XRD, Bruker D8 Focus Powder XRD) analysis. X-ray photoelectron spectroscopy (XPS) was conducted with an X-ray photoelectron spectroscope (ESCALAB 250 XI, Thermo Fisher Scientific, Waltham, MA, USA).To further investigate its physical properties, dynamic light scattering (DLS) technology from Malvern, UK was used to accurately quantify its zeta potential and particle size distribution.

### 2.4 Drug loading and release

After a careful rinsing procedure with methyl alcohol to remove any unencapsulated lithospermic acid, LA@ZIF-8 nanoparticles were successfully synthesized. A certain amount of dry LA@ZIF-8 nanoparticles is taken and denoted as m_0_. Subsequently, the nanoparticles are completely dissolved using a dilute nitric acid solution, then centrifuged and filtered, where the residue represents the extracted LA, LA was freeze-dried and weighed as m_L_.The loading ratio (LR) = m_L_/m_0_∗100 %. The lithospermic acid release profile was studied under acidic (pH 6.0) and neutral (pH 7.2) conditions. Initially, 1 mL of LA@ZIF-8 nanoparticles was encapsulated in a dialysis membrane (MWCO, 3500 Da). This membrane was immersed in 30 mL of phosphate buffered saline (PBS), supplemented with 5 % polysorbate 80, at the specified pH levels. The system was shaken on a vibrating table at a constant speed of 100 rpm and maintained at a constant temperature of 37 °C. At predetermined time intervals (0, 0.5, 1, 2, 4, 6, 8, 12, 24h), PBS was removed for analysis and refreshed with fresh PBS. The concentrations of lithospermic acid released in the PBS samples were accurately determined by UV–visible spectroscopy at 370 nm.

### 2.5 Cell culture and treatment

RAW264.7 cells were grown in DMEM medium supplemented with 10 % fetal bovine serum and 1 % penicillin-streptomycin only. RAW264.7 cells are induced into M1-type macrophages after 12 h of induction with 100 ng/mL LPS. Cells grown in fresh medium were used as a control. The ATDC5 cell line was cultured in DMEM/F12 medium supplemented with 5 % (v/v) fetal bovine serum (FBS) and 1 % penicillin-streptomycin solution at 37 °C in a humidified incubator maintained at 5 % CO_2_. To stimulate ATDC5 cell differentiation, 1 % (v/v) insulin-transferrin-selenium (ITS) supplement was used. After an incubation period of 96 h, a sophisticated pattern of cell proliferation, indicative of differentiation, materialized.

### 2.6 Macrophages conditioned medium (CM) collection

RAW264.7 cells were seeded at a density of 1 × 10^6^ cells/well in a 6-well plate and then incubated with 100 ng/mL LPS plus 50 μg/mL LA, 50 μg/mL ZIF-8 or 50 μg/mL LA@ZIF-8 for 12 h, cells cultured in fresh medium were used as control. After that, the supernatant of each group was centrifuged at 1000 g for 5 min and stored at −80 °C for further experiments. CM was diluted with serum-free DMEM-F12 medium to a final concentration of 50 % and added to Petri dishes for chondrocyte culture [31].

### 2.7 In vitro cytotoxicity

Cell cytotoxicity was assessed using the Cell Counting Kit-8 (CCK-8). Briefly, Raw264.7 cells were cultured in 96-well plates at a density of 1 × 10^4^ cells/well, followed by LA treatments (0, 12.5, 25, 50, 100, 200 or 400 μg/mL),ZIF-8 treatments (0, 12.5, 25, 50, 100, 200 or 400 μg/mL) or LA@ZIF-8 treatments (0, 12.5, 25, 50, 100, 200 or 400 μg/mL) for 24 h. Each plate was then filled with 100 μl of 10 % CCK-8 solution and incubated at 37 °C for 120 min. The absorbance of the wells was measured at 450 nm using a microplate reader (Leica Microsystems, Wetzlar, Germany). All results are received through three independent experiments.

### 2.8 Intracellular uptake of nanoparticles

Endocytosis was monitored using LA@ZIF-8 for fluorescence localization. LA@ZIF-8 was incubated with RAW264.7 cells in a 6-well plate. After a 12 h incubation, the cells were washed with PBS and then incubated with DAPI. Finally, the slides were examined under a confocal laser scanning fluorescence microscope (CLSM).

### 2.9 Therapeutic effect on M1 polarization of RAW264.7

As described before, LPS has been used to simulate synovitis in the development of osteoarthritis. RAW264.7 cells were divided into four groups: 1) control group: RAW264.7 treated with fresh medium only; 2) LPS group: RAW264.7 cells induced in macrophages M1-type modeled after 12 h induction with 100 ng/mLLPS; 3)ZIF-8 group: RAW264.7 was incubated with 100 ng/mL LPS plus 50 μg/mL ZIF-8 for 12 h. 4) LA group: RAW264.7 was incubated with 100 ng/mL LPS plus 50 μg/mL LA for 12 h; 5) LA@ZIF-8 group: RAW264.7 was incubated with 100 ng/mL LPS plus 50 μg/mL LA@ZIF-8 for 12 h.

### 2.10 ROS detection

After 12 h of culture in 6-well plates, ROS levels in different groups of RAW264.7 cells were detected using the fluorescence probe DCFH-DA. The cells were observed under a fluorescence microscope (DM4000 B; Leica, Germany).

### 2.11 Western blotting

RAW264.7 cells or ATDC5 chondrocytes were seeded in 6-well plates to collect total protein. RAW264.7 cells were stimulated with LPS (100 ng/mL) in the presence or absence of LA (50 μg/mL), ZIF-8 (50 μg/mL) or LA@ZIF-8 (50 μg/mL) treatment for 12 h. CM was co-cultured with ATDC5 chondrocytes for 24 h. RAW264.7 cells or ATDC5 chondrocytes were lysed with radioimmunoprecipitation lysis buffer (RIPA) containing a cocktail of protease and phosphatase inhibitors. Quantification of lysates was evaluated with a BCA (bicinchoninic acid) test kit before its denaturation process at 99 °C for a duration of 10 min. The samples were transferred to nitrocellulose filter membranes (Millipore, USA). For the detection of specific proteins, target antibodies are used during the incubation process. Proteins were visualized and documented using BIO-RAD's ChemiDOC Western blot imaging system. Antibodies used for western blotting were TNF-α, IL-1β, iNOS, COX-2, GAPDH, Col2a1, Mmp13, Phospho-NF-κB, NF-κB, IKKβ, p-IKKβ, Sox9, Mfn, Opa, Fis1, Drp1. GAPDH was used as an internal control.

### 2.12 Quantitative real–time PCR

RAW264.7 or ATDC5 cells were plated in 6-well plates to collect total RNA. After treatment in each group, the TRIzol™ Plus RNA purification kit was used to lyse RAW264.7 or ATDC5 cells according to the manufacturer's instructions. Complementary cDNA was synthesized by PrimeScript™ RT Master Mix and real-time PCR analyzes were performed in QuantStudio 7 (Thermo) using Hieff® qPCR SYBR Green Master Mix. The cDNA was predenatured at 95 °C for 2 min and denatured at 95 °C for 10 s and annealing/stretching at 60 °C for 30s. The primers used were the following: β-actin, forward, 5′-TATGCTTCCCTCACGCCATCC-3'; reverse, 5′-GTCACGCACGATTTCCCTCTCTCAG-3'; IL-1β, forward, 5′-TCCGAGCAGCACATCAACAAGAG-3'; reverse, 5′-AGGTTCCACGGGAAAGACACAGG-3'; COX-2, forward, 5′-TTCAACACACTCTATCACTGGC-3′, reverse, 5′-AGAAGCGTTTGCGGTACTCAT-3'; iNOS, forward, 5′-GTTCTCAGCCCAACAATACAAGA3′, reverse, 5′-GTGGACGGTCGATGTCAC3'; Sox9, forward, 5′-GAGCCGGATCTGAAGAGGGA-3'; reverse, 5′-GCTTGACGTGTGGCTTGTTC-3'; Mmp9, forward, 5′-CGCCACCACAGCCAACTATGACA-3′ reverse, 5′-CTGCTTGCCCAGGAAGACGAAGA-3'.Tnf-α, forward, 5′ GACGTGGAACTGGCAGAAGAG-3′ reverse, 5′-TTGGTGGTTTTGTGAGTGTGAG-3'; Adamts5, forward, 5′-CAACAGGAGGATCATCGCAGATACAG-3'; reverse, 5′-CCAAGGTCACCATCATTACACCAAGT-3'; Mmp13, forward, 5′-TGGAGTAATCGCATTGTGAGAGTC-3′, reverse, 5′-CCAGCCACGCATAGTCATATAGATAC -3'. Expression levels of specific RNAs were normalized to β-actin.

### 2.13 Immunofluorescence of RAW264.7 and ADTC5 in vitro

LPS-treated RAW264.7 cells were co-cultured with LA (50 μg/mL),ZIF-8 (50 μg/mL)or LA@ZIF-8 (50 μg/mL)in 6-well plates for 12 h. CM was co-cultured with ATDC5 chondrocytes for 24 h. Then, the cells were washed three times with PBS and fixed with a 4 % paraformaldehyde solution. After incubating with 0.5 % Triton X-100 in PBS for 15 min, the cells were blocked with 5 % goat serum for 1 h. Cells were then treated with primary antibodies against Tnf-α, iNOS, Col2a1 and Mmp13 applied at 4 °C overnight. Secondary antibodies were incubated for 60 min at room temperature with Alexa Fluor 555 label or Alexa Fluor 488 label. Images were examined by fluorescence microscopy after 1 min of DAPI labeling.

### 2.14 Alcian blue staining of ATDC5 in vitro

Alcian blue staining of ATDC5 cells in 6-well plates was performed to visualize cartilage degeneration. ATDC5 cells were cultured in 6-well plates overnight. Following a 24-h treatment with CM, the cells were stained with Alcian blue according to the manufacturer's recommended protocol. Finally, images were analyzed using a light microscope.

### 2.15 Mitochondrial membrane potential (JC-1) analysis

After culture for 24 h with CM in 6-well plates, ATDC5 chondrocytes were washed once with PBS, 1 mL of cell culture medium and 1 mL of JC-1 staining medium were added and cultivated at 37 °C for 20 min. Remove the supernatant and wash three times with JC-1 staining buffer. By adding 2 mL of cell medium, the cells were observed under a confocal laser microscope [32].

### 2.16 Mitochondrial morphology analysis

After 24 h of culturing ATDC5 chondrocytes with CM in 6-well plates, mitochondrial morphology was assessed using the aforementioned methodologies and reagents. Then, Mito-Tracker Green (1:5000) and Mito-Tracker Red CMXRos (1:1000) solution were introduced and incubated at 37 °C for 30 min. The MitoTracker Red CMXRos solution was then discarded and fresh cell culture medium prewarmed to 37 °C was added. The cells were then examined using the laser confocal microscope (TCS SP8; Leica, Germany).

### 2.17 Transmission electron microscopy (TEM) analysis

ATDC5 chondrocyte cells after treatment in 6-well plates were examined using TEM. Adherent cell blocks, obtained by centrifugation, were subjected to fixation and dehydration procedures. Specifically, the sample was initially fixed with 2.5 % glutaraldehyde for a duration of 2.5 h, followed by three washes in phosphate buffered saline (PBS) (0.1 M, pH 7.0), each lasting 3 min and fixed in 1 % tetroxidedosmium for 2 h. After that, the cells are washed three times.

Cells were subjected to a continuous dehydration process using a graded ethanol series, followed by two cycles of dehydration in a 1:1 mixture of ethanol and acetone, and finally in pure acetone. Each time, the cells were infiltrated with acetone: resin 3:1, 1:1, 1:1, 1:1, 1:1 and 1:1 for 1 h. Cells were impregnated with resin overnight and embedded in fresh resin for 3 h. After an additional polymerization at 37 °C for 8 h and at 65 °C for 48 h, The resin segments were sliced into ultrathin slices (70–100 nm). Each ultrathin tissue section was precisely placed on a copper grid with carbon film and then stained with uranyl acetate at 4 °C for 7 min. After staining, slices were carefully cleared, followed by a second lead citrate stain at 25 °C for 3 min, then observed and images captured (Talos L120C, FEI; Thermo Fisher Scientific).

### 2.18 Establishment of anterior cruciate ligament transection (ACLT) in mice

24 male C57BL/6 mice, 8 weeks old, were obtained from Shanghai SIPPR BK Laboratory Animals Ltd. (Shanghai, China) for the study. Mice were maintained under a 12 h light/dark cycle with ad libitum access to food and water. All animals used in this study received ethical approval and were cared for in accordance with the institutional guidelines established by the Experimental Animal Ethics Committee of Shanghai Jiao Tong University School of Medicine (XHEC-F-2025-003). Osteoarthritis (OA) models were created by the ACLT method, divided into the sham operation group, the ACLT group,the ACLT + ZIF-8 group, the ACLT + LA group and the ACLT + LA@ZIF-8 group. Five days after surgery, intra-articular injections of LA, ZIF-8 or LA@ZIF-8 were administered for subsequent histological analysis. Speciﬁcally, mice in ACLT group were injected intra-articularly with PBS, and mice in ACLT + ZIF-8 group and ACLT + LA@ZIF-8 were injected intra-articularly with 100 μL of 50 μg/mL ZIF-8 or LA@ZIF-8 NPs, and mice in LA group were injected intra-articularly with 100 μL of 50 μg/mL LA every 3 days for 4 weeks.

### 2.19 In vivo imaging system (IVIS) imaging

The residence time and biodistribution behaviors of LA@ZIF-8 were observed using a noninvasive IVIS (AniView 100, BLT, China) by the administration of NPs into OA mice intra-articularly. After 72 h, specific tissues, such as the heart, spleen, lung, liver, and kidney, were obtained for IVIS.

### 2.20 Histological and immunofluorescence analyses and OARSI scoring

The knee joints of mice with osteoarthritis (OA) were collected and then fixed in 4 % paraformaldehyde (PFA). The samples were decalcified with 10 % EDTA before being embedded in paraffin. Safranin O-fast green and hematoxylin and eosin (H&E) staining were used to assess changes in cartilage microstructure after LA, ZIF-8 or LA@ZIF-8 treatment in 4-μm knee sections. As mentioned, the Mankin score and the International Osteoarthritis Research Society score (OARSI) were calculated. Immunohistochemical (IHC) staining was performed with antibodies against iNOS, p-P65, Col2a1, Aggrecan, Sox9. Immunofluorescence staining was performed with an antibody against iNOS, Drp1. The percentage of positively stained cells or positive field in synovium and articular cartilage was determined by Image J software. In addition, to evaluate the systemic side effect of nanoparticles, the main organs of a subgroup of mice, especially the heart, spleen, lungs. liver and kidney, were removed. To confirm the therapeutic effect roles of LA@ZIF-8 in vivo.

### 2.21 Statistical analysis

SPSS 20.0 software was used for this statistical analysis. All quantitative data sets are presented as mean ± standard deviation (SD). Differences between groups were analyzed by one-way analysis of variance (ANOVA), followed by Tukey's post hoc test. Statistical significance is considered ∗ p < 0.05, ∗∗p < 0.01, ∗∗∗p < 0.001.

## 3 Results

### 3.1 LA@ZIF-8 synthesis and characterisation

**Fig. 1:**
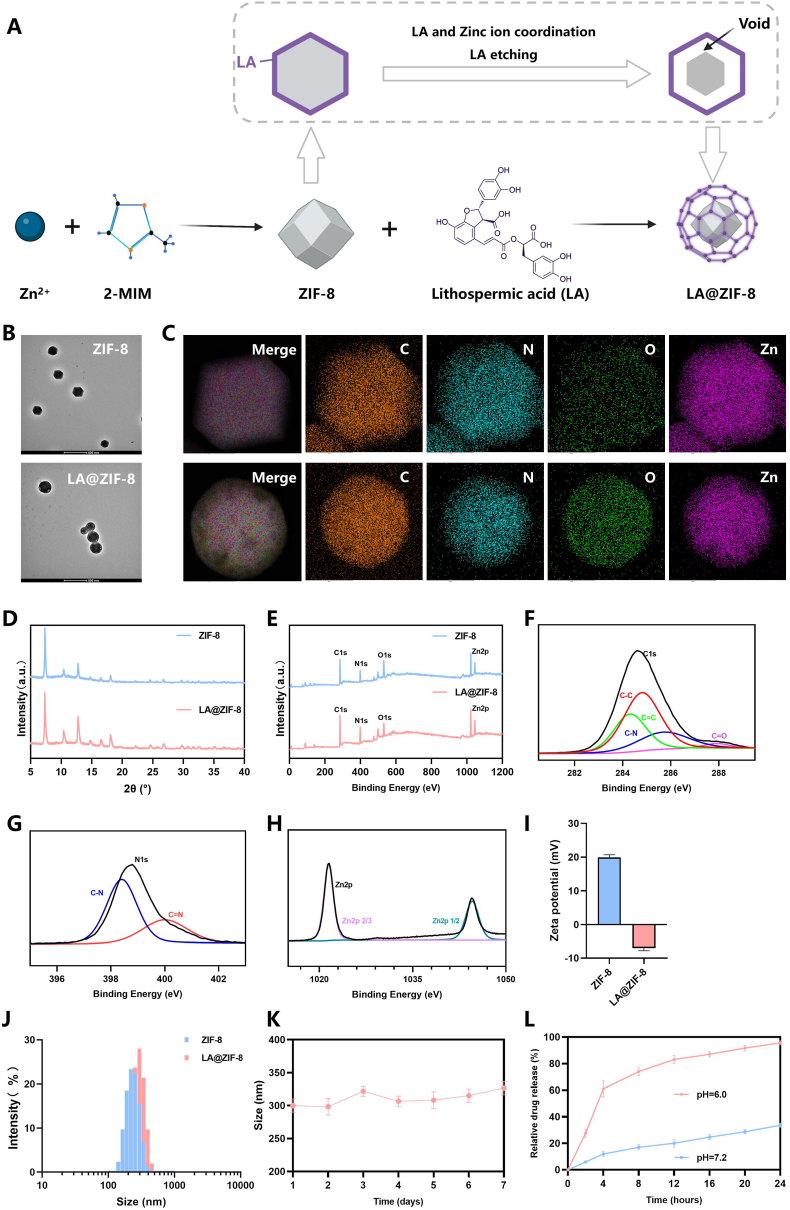
**Synthesis and characterization of LA@ZIF-8.** A) LA etching ZIF8 synthesis process and mechanism diagram. B) TEM images of ZIF-8 and LA@ZIF-8. C) Mapping of ZIF-8 and LA@ZIF-8. D) XRD images of ZIF-8 and LA@ZIF-8. E) XPS of ZIF-8 and LA@ZIF-8 for full survey scan. F) XPS analyses of C 1s in LA@ZIF-8. G) XPS analyses of N 1s in LA@ZIF-8. H) XPS analyses of Zn2p in LA@ZIF-8. I) Zetal potential of ZIF-8 and LA@ZIF-8. J) DLS of ZIF-8 and LA@ZIF-8. K) The stability analysis of LA@ZIF-8. L) Cumulative in vitro drug release of LA@ZIF-8 in PBS (pH 6.0 and 7.2). The data are presented as the mean ± SD. n = 3.

LA etching ZIF-8 synthesis process and mechanism diagram (Fig. 1A),LA, as a polyphenolic compound featuring phenolic hydroxyl groups that act as chelating sites, can coordinate with zinc ions on the outside of ZIF-8 particles. This coordination facilitates the release of free H+ ions, which attach to LA molecules and subsequently infiltrate into ZIF-8, achieving gradual internal etching. Due to the strong metal-phenol interaction, a large amount of LA in the solution tends to adhere to the surface of ZIF-8, stabilising the overall crystal form of ZIF-8 and promoting the transformation of the surface of ZIF-8 from hydrophobic to hydrophilic. TEM images clearly show that the ZIF-8 particles are ZIF-8 NPs has a uniform, monodisperse hexagonal morphology and a smooth surface, with a size of about 200–300 nm. After LA modification, the surface of ZIF-8 particles became rough, and small voids were created within each ZIF-8 particle and a network structure appeared, confirming that protons were dissociated from LA and etched inward through the hydrophilic surface of ZIF-8 (Fig. 1B). The distribution of O elements is more dense than that of ZIF-8 particles, possibly due to the large amount of phenol hydroxyl group contained in LA.Compared to the element distribution map of ZIF-8 particles, there are notable areas where elemental distribution appears absent (Fig. 1C). The XRD characterization verifies the structure of LA-modified ZIF-8, as evidenced by characteristic diffraction signals that match those of pristine ZIF-8 crystals, demonstrating the preservation of framework topology during LA incorporation(Fig. 1D). The XPS survey spectrum of the LA@ZIF-8 revealed the presence of O, C, N, and Zn(Fig. 1E). The XPS data of C 1s spectrum of the LA@ZIF-8 showed typical types of carbon bonds: C-N, C=O, C-C and C=C respectively(Fig. 1F). N 1s indicated typical types of carbon bonds: C-N and C=N (Fig. 1G). Zn 2p indicated typical types of bonds: Zn2p 2/3 and Zn2p 1/2(Fig. 1H). After LA etching, the zeta potential of ZIF-8 decreased from 19 to −7 mV, possibly due to the large number of phenolic hydroxyl groups on the surface of ZIF-8, further confirming the successful preparation (Fig. 1I). LA@ZIF-8 has a slightly larger diameter compared to ZIF-8, possibly due to the coordination of LA on the surface of ZIF-8 with zinc ions, forming a metal-phenol network (Fig. 1J). The particle size of LA@ZIF-8 varies slightly between 291 and 317 nm, indicating excellent stability over 7 days (Fig. 1K). In order to investigate the pH response behavior of ZIF-8 NPs, in vitro release studies of LA were conducted in PBS with different pH values (pH6.0, pH 7.2), Under pH 7.2 conditions, the drug release rate of LA was only 19.8 % at 12 h and 37.2 % at 24 h. In contrast, under pH 6.0 conditions, the cumulative drug release rate was significantly accelerated, reaching 81.2 % at 12 h and 96.2 % at 24 h (Fig. 1L). These findings indicate that LA@ZIF-8 exhibits a responsive behavior in mildly acidic environments, thereby enabling controlled release of LA. These results suggest that LA@ZIF-8 responds to mildly acidic environments and allows for controlled release of LA.

### 3.2 Cytotoxicity and cellular uptake of LA@ZIF-8

**Fig. 2:**
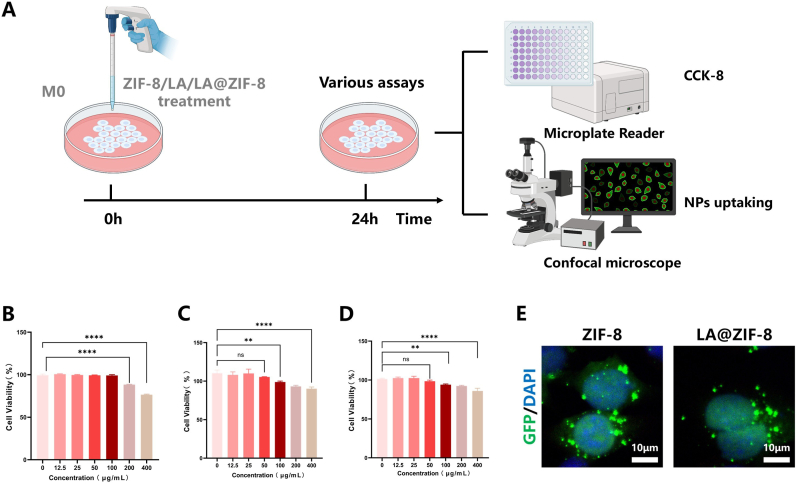
**Evaluate the impact of LA@ZIF-8 on M0 macrophages.** A) Schematic diagram to explore the safe concentration of ZIF-8, LA and LA@ZIF-8. B-D) CCK8 assay of macrophages treated with various concentrations of ZIF-8, LA and LA@ZIF-8 for 24h. E) Fluorescence images of RAW264.7 cells endocytosis with ZIF8 and LA@ZIF-8 for 24 h. Scale bars are 25 μm. The data are presented as the mean ± SD. ∗P < 0.05, ∗∗P < 0.01, ∗∗∗P < 0.001, n = 3.

To determine the safe concentrations of LA and LA@ZIF-8 acting on RAW264.7 cells, we conducted a co-incubation experiment with ZIF-8, LA or LA@ZIF-8 for 24 h (Fig. 2A). The effect of ZIF-8,LA and LA@ZIF-8 on RAW264.7 cells viability was measured using the CCK-8 assay. When the concentration of ZIF-8 reaches 200 μg/mL, it will produce obvious toxic effect on raw264.7 cells, indicating that ZIF-8 has high biocompatibility (Fig. 2B). The results indicated that LA had no significant cytotoxic effect on RAW264.7 cells in the concentration range of 0–50 μg/mL. However, the cells viability decreased significantly when the LA concentration exceeded 50 μg/mL (Fig. 2C). The obvious toxicity of LA@ZIF-8 was observed only when the concentration of LA@ZIF-8 reached 100 μg/mL., (Fig. 2D). Consequently, a concentration of 50 μg/mL was selected for subsequent experiments on LA and LA@ZIF-8. GFP was encapsulated in ZIF-8 and LA@ZIF-8 and co-incubated with RAW264.7 cells for 24 h. Confocal laser microscopy revealed green fluorescence localised around the nucleus, confirming cellular uptake of ZIF-8 and LA@ZIF-8 (Fig. 2E).

### 3.3 LA@ZIF-8 suppressed LPS-induced M1 polarization of RAW264.7 macrophages

**Fig. 3:**
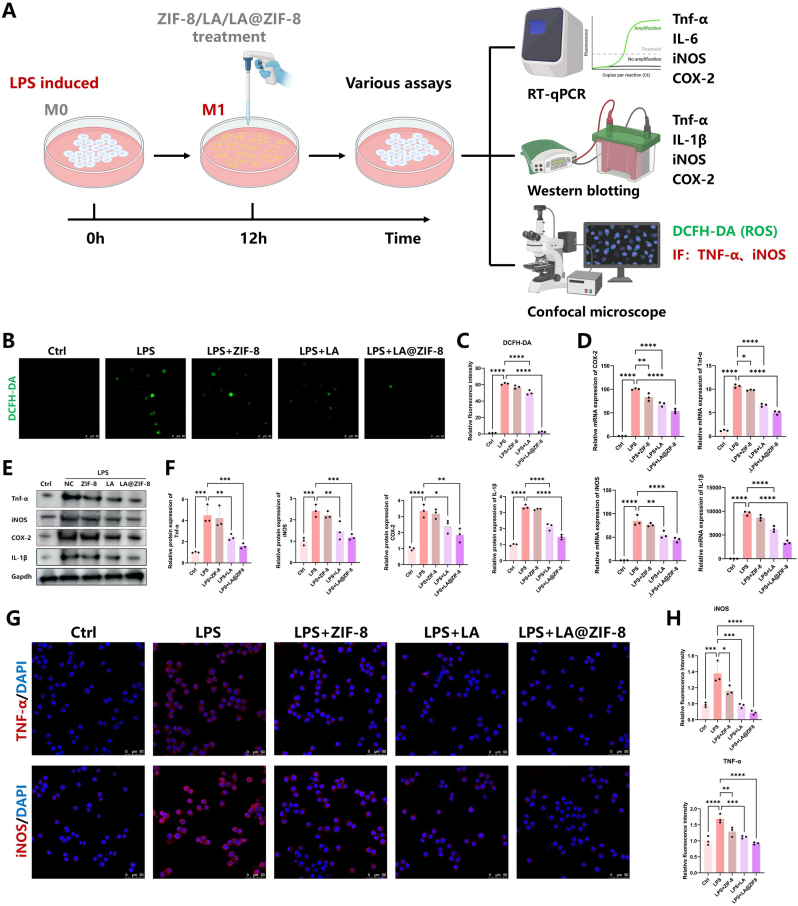
**Effect of LA@ZIF-8 on anti-inflammatory in LPS-activated RAW264.7 cells.** A) Schematic diagram of explore the effect of ZIF-8, LA and LA@ZIF-8 on M1 macrophage inflammation. B) Representative immunofluorescence images of LPS-stimulated RAW264,7 cells stained with ROS fluorescent probe DCFH-DA. Scale bars are 50 μm. C) Quantitative analysis of ROS level. D) Expression of M1 macrophage markers COX-2, Tnf-α, iNOS and IL-1β were examined by qPCR. E) Western blot showing the expression of Tnf-α, iNOS, COX-2 and IL-1β in macrophages after various treatments. F) Quantification of expression levels of Tnf-α, iNOS, COX-2 and IL-1β protein. G) Representative images of immunostaining for M1 marker (TNF-α and iNOS) in RAW264.7 cells in the different groups. Scale bars are 50 μm. H) Quantification of the fluorescence intensity in different groups. The data are presented as the mean ± SD. ∗P < 0.05, ∗∗P < 0.01, ∗∗∗P < 0.001, n = 3.

RAW264.7 cells were treated with 100 ng/mL LPS to induce M1 macrophages, establishing a model of arthritic synovitis cells, followed by treatment with LA or LA@ZIF-8. Therapeutic effects were evaluated based on the expression of inflammation-related genes or proteins (Fig. 3A). The inflammatory factors produced by M1 macrophages can cause mitochondrial dysfunction, leading to oxidative stress and ROS accumulation. The macrophages in each group were labeled with DCFH-DA to evaluate the effects of LA and LA@ZIF-8 on ROS clearance by M1 macrophages. LPS-treated M1 macrophages produced a substantial amount of ROS, and the green fluorescence intensity was significantly reduced following LA and LA@ZIF-8 treatments. LA@ZIF-8, on the other hand, showed a stronger ability to clear ROS (p < 0.0001) (Fig. 3B and C). RAW264.7 macrophages induced by LPS were treated with 50 μg/mL LA, 50 μg/mL ZIF-8 or 50 μg/mL LA@ZIF-8, and the mRNA expression levels of M1 macrophage markers TNF-α, IL-1β, iNOS, and COX-2 were measured in each group. The expression of inflammation-related genes in RAW264.7 macrophages was significantly downregulated following treatment with LA or LA@ZIF-8 (Fig. 3D). Further analysis of inflammation-related protein expression in macrophages revealed significant upregulation of TNF-α, iNOS, COX-2, and IL-1β in RAW264.7 cells in the LPS group. Treatment with LA or LA@ZIF-8 downregulated these protein levels, with LA@ZIF-8 showing stronger anti-inflammatory effects (Fig. 3E and F). M1-type macrophage markers, TNF-α and iNOS, were labeled by immunofluorescence staining. Following treatment in the LA and LA@ZIF-8 groups, the level of M1 macrophage markers, TNF-α, and iNOS, were significantly decreased compared to those in the LPS group, demonstrating strong anti-inflammatory effects. The efficacy of LA was enhanced in the LA@ZIF-8 group (Fig. 3G and H). In conclusion, LA@ZIF-8 inhibited the LPS-induced pro-inflammatory phenotype of RAW264.7 macrophages and effectively reduced ROS accumulation caused by inflammation.

### 3.4 LA@ZIF-8 salvage inflammation-induced mitochondrial damage in RAW264.7 macrophages

**Fig. 4:**
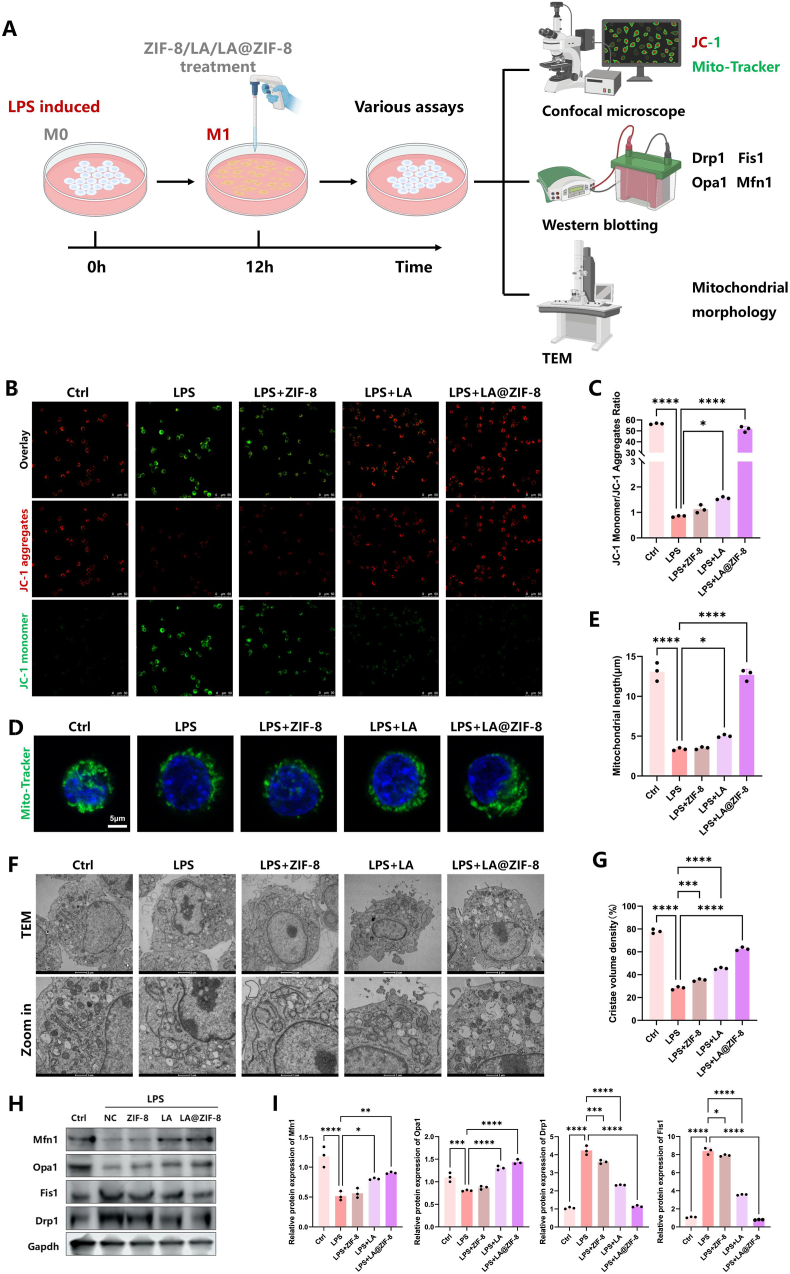
**Mitochondrial Function Recovery by LA@ZIF-8** A) Schematic diagram of mechanism by which LA@ZIF-8 reduces mitochondrial dysfunction in M1-polarized macrophages. B) Representative images of Mitochondrial membrane potential of M1 macrophages after various treatments stained with JC-1. Scale bars are 50 μm. C) Quantitative analysis of JC-1 monomer/JC-1 aggregates ratio. D) RAW264.7 cells were treated with various treatment incubation with or without LPS and stained with Mito-Tracker observe mitochondrial function. E) Quantitative analysis of mitochondrial length. F) Mitochondrial morphology observed by TEM. G) Quantitative analysis of mitochondrial cristae volume density. H) The protein expression of Mfn1, Opa1, Fis1 and Drp1 in RAW264.7 cells after various treatments. I) Quantification of expression levels of Mfn1, Opa1, Fis1 and Drp1 protein. The data are presented as the mean ± SD. ∗P < 0.05, ∗∗P < 0.01, ∗∗∗P < 0.001, n = 3.

To further verify the therapeutic effects of LA, ZIF-8 and LA@ZIF-8 on mitochondrial dysfunction in LPS-induced M1-polarized macrophages, RAW264.7 cells were treated with 100 ng/mL LPS. The mitochondrial membrane potential, mitochondrial morphology, and related protein expression levels were compared before and after LA or LA@ZIF-8 treatment in all groups (Fig. 4A). Fluorescence staining revealed that the mitochondrial membrane potential of macrophages was significantly reduced after LPS stimulation, indicating that LPS induces mitochondrial dysfunction in M1 macrophages. After treatment with LA, ZIF-8 or LA@ZIF-8, the red fluorescence of macrophage mitochondrial polymers significantly increased, while the green fluorescence of mitochondrial monomers significantly decreased (Fig. 4B and C). Mitochondria are highly dynamic, with their form and function regulated through fusion and division. Excessive mitochondrial division leads to fragmentation and mitochondrial network damage. Mitochondrial probes were used to label the macrophage mitochondria in each group, and LA@ZIF-8 effectively reversed LPS-induced mitochondrial division in macrophages (Fig. 4D and E). The morphology of mitochondria was observed using TEM, revealing oval or oblong cross-sections with regular ridge arrangements in normal mitochondria. Following LPS stimulation, the mitochondrial matrix swelled, and the ridge density decreased. Mitochondrial damage in each group was characterized by the mitochondrial cristae volume density analysis [10,12]. After treatment with LA@ZIF-8, mitochondrial matrix swelling and ridge disorders were significantly reduced (Fig. 4F and G). The expression levels of mitochondrial division and fusion proteins Drp1, Fis1, Opa1, and Mfn1 were analyzed. It was observed that the levels of Drp1 and Fis1 proteins associated with mitochondrial division significantly increased in M1 macrophages after LPS induction, while the levels of Opa1 and Mfn1 proteins associated with mitochondrial fusion significantly decreased (Fig. 4H and I). These results indicate that LPS-induced mitochondrial division was predominant in M1 macrophages. Compared to the LPS group, the protein levels of Drp1 and Fis1 were significantly downregulated following treatment with LA or LA@ZIF-8, while the protein levels of Opa1 and Mfn1 were significantly upregulated. This suggests that LA or LA@ZIF-8 treatment had an inhibitory effect on LPS-induced mitochondrial hyperdivision in M1 macrophages. In conclusion, the treatment of mitochondrial dysfunction with ZIF-8 alone has no effective therapeutic effect. LA@ZIF-8 significantly alleviated LPS-induced mitochondrial dysfunction of RAW264.7 macrophages and markedly improved mitochondrial morphology; its efficacy was superior to that of LA alone.

### 3.5 LA@ZIF-8 protects ATDC5 chondrocytes by inhibiting the polarization of RAW264.7 macrophages

**Fig. 5:**
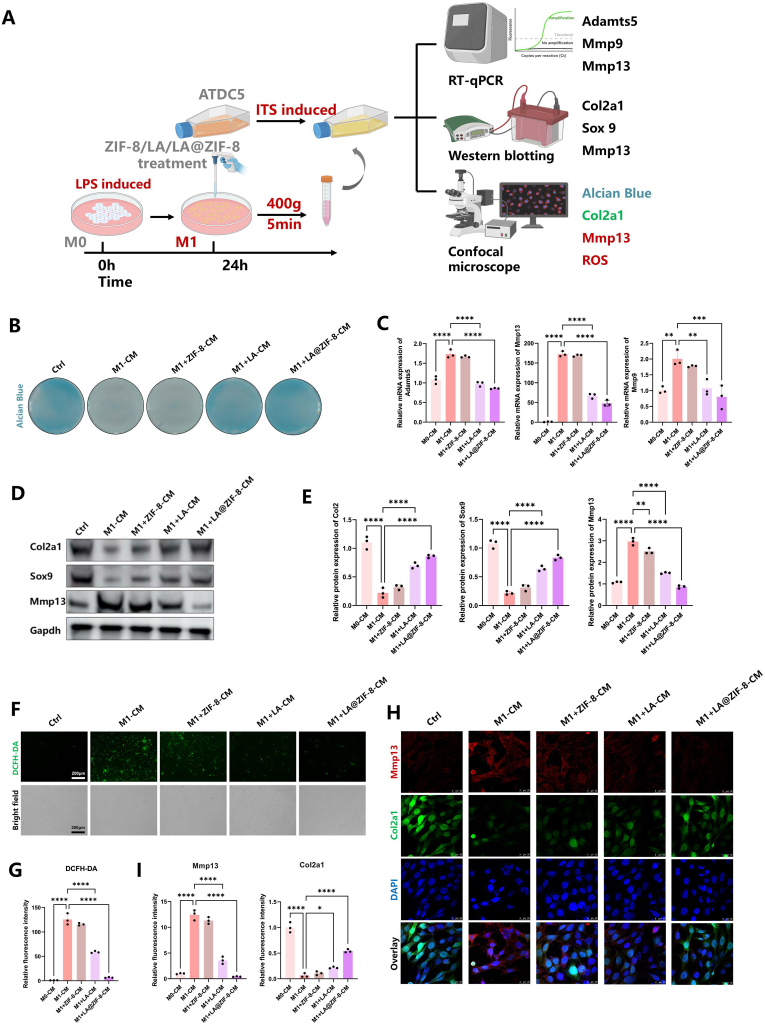
**Protective effect of LA@ZIF-8 on chondrocytes.** A) Schematic diagram of the CMs was co-cultured with ATDC5 induced by ITS to verify the protective effect of LA@ZIF-8-CM on chondrocytes. B) Alcian blue staining of mucopolysaccharide (accharide) -like proteoglycan in ATDC5 chondrocytes. C) The catabolic genes(Adamts5、Mmp9、Mmp13) were identified by qPCR. D) The protein expression of synthesis and decomposition of related protein (Col2a1, Sox9 and Mmp13) in ATDC5 chondrocytes after various treatments. E) Quantification of expression levels of Col2a1, Sox9 and Mmp13 protein. F) Representative immunofluorescence images of ATDC5 chondrocytes after different treatments stained with ROS fluorescent probe DCFH-DA. Scale bars are 200 μm. G) Quantitative analysis of ROS. H) Representative images of immunostaining for Mmp13 and Col2a1 in ATDC5 chondrocytes after different treatments. I) Quantification of the fluorescence intensity in different groups. The data are presented as the mean ± SD. ∗P < 0.05, ∗∗P < 0.01, ∗∗∗P < 0.001, n = 3. (For interpretation of the references to colour in this figure legend, the reader is referred to the Web version of this article.)

To assess the effect of M1 macrophages on chondrocytes, the CM of treated RAW264.7 cells was incubated with ATDC5 chondrocytes, which were induced by ITS for 24 h. The synthesis and degradation function of the chondrocytes were then evaluated (Fig. 5A). After 24 h of incubation, the M1+LA-CM and M1+LA@ZIF-8-CM groups experienced reversed dysregulation of chondrocyte synthesis and catabolic homeostasis caused by M1-CM, but ZIF-8 alone has no therapeutic effect (Fig. 5B). Expression of the catabolism-related genes/proteins Adamts5, Mmp9, and Mmp13 in the M1-CM group was significantly reduced, while the expression of the metabolism-related proteins Col2a1 and Sox9 significantly increased (Fig. 5C–E). DCFH-DA was used to label each group to evaluate the effect of different CMs on ROS production in ATDC5 chondrocytes. Results indicated that ATDC5 chondrocytes treated with M1-CM produced more ROS, and using ZIF-8 alone does not reduce ROS generation. LA and LA@ZIF-8 significantly reversed chondrocyte ROS accumulation induced by M1-CM (Fig. 5F and G). Immunofluorescence analysis confirmed that Mmp13 expression significantly increased, while Col2a1 expression significantly decreased in ATDC5 chondrocytes treated with M1-CM, M1+LA-CM and M1+LA@ZIF-8-CM groups significantly reversed this phenomenon (Fig. 5H and I). In summary, LA@ZIF-8 has stronger protection against macrophage inflammation in OA chondrocytes compared with LA.

### 3.6 LA/LA@ZIF-8 suppress inflammation in RAW264.7 macrophages by inhibiting the NF-κB signaling pathway

**Fig. 6:**
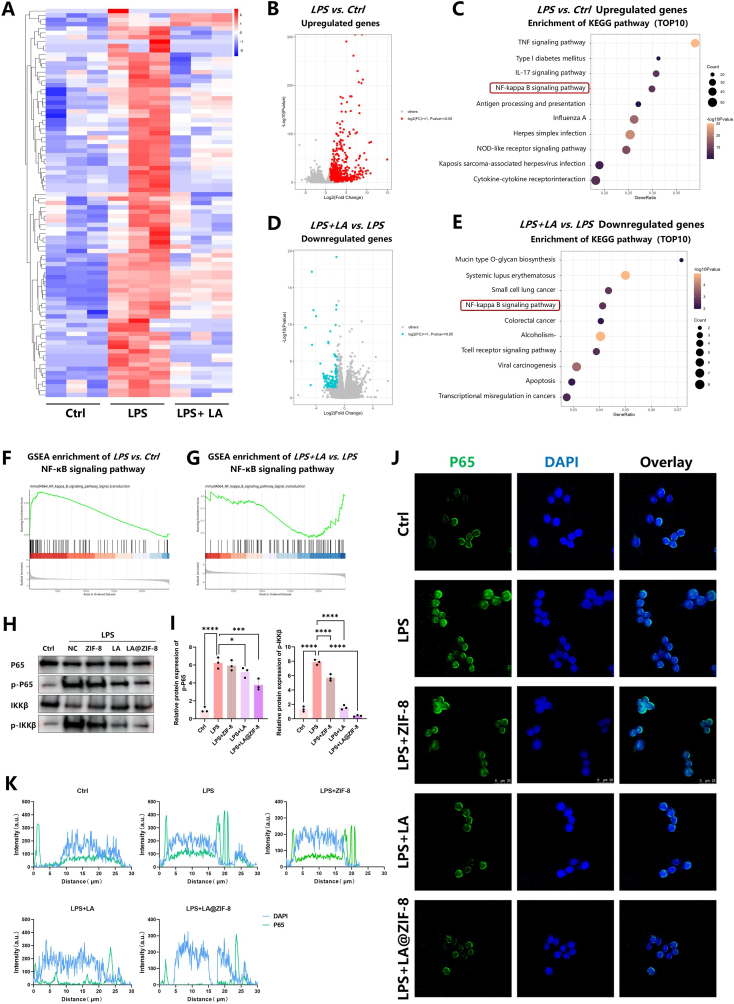
**RNA-seq analysis of the anti-inflammatory mechanisms of LA@ZIF-8.** A) Heat map of RNA-seq by Ctrl, LPS and LPS + LA. B) Volcano plot representation of differentially expressed genes between LPS and Ctrl group (adjusted 1.5 fold, p-value <0.05). C) KEGG pathway enrichment analysis of differentially expressed genes in LPS group and Ctrl group. D) Volcano plot representation of differentially expressed genes between LA + LPS and LPS group (adjusted 1.5 fold, p-value <0.05). E) KEGG pathway enrichment analysis of differentially expressed genes in LPS group and LA treatment group. F) GSEA analysis showing differentially expressed genes in NF-κB in Ctrl group and LPS group. G) GSEA analysis showing differentially expressed genes in NF-κB in LPS group and LPS + LA group. H) The protein expression of P65, p-P65, IKKβ and p-IKKβ protein. I) Quantification of expression levels of P65, p-P65, IKKβ and p-IKKβ protein. J) Representative images of immunostaining for P65. K) Nuclear and P65 colocalization analysis. The data are presented as the mean ± SD. ∗P < 0.05, ∗∗P < 0.01, ∗∗∗P < 0.001, n = 3.

To explore the molecular mechanism of LA/LA@ZIF-8 in reducing OA synovial macrophage inflammation, we performed genome-wide RNA-sequencing on LPS-stimulated RAW264.7 cells treated with LA (Fig. 6A). Kyoto Encyclopedia of Genes and Genomes enrichment analysis showed that the differentially expressed genes, which were significantly upregulated after LPS treatment, were mainly involved in inflammatory signaling pathways, including NF-κB (Fig. 6B and C). The inflammatory signaling pathways were significantly inhibited by LA treatment (Fig. 6D and E). Gene Set Enrichment Analysis (GSEA) showed that the NF-κB signaling pathway in RAW264.7 macrophages was significantly upregulated after LPS induction, while it was downregulated after LA treatment (Fig. 6F and G). These results suggest that LA may exert its anti-inflammatory role by inhibiting NF-κB signaling activation. To further verify the anti-inflammatory mechanism of LA, we measured the expression levels of proteins related to the NF-κB signaling pathway. The results indicated that the phosphorylation levels of P65 and IKKβ were significantly increased after LPS treatment of RAW264.7 cells, while they were significantly decreased after treatment with LA or LA@ZIF-8. Moreover, the degree of decline was more pronounced in the LA@ZIF-8 group, ZIF-8 nanoparticles alone could not effectively inhibit the activation of P65 and IKKβ (Fig. 6H and I). Immunofluorescence images of p-P65 further demonstrated the phosphorylation level of P65 (Fig. 6J), and the nuclear translocation of P65 in RAW264.7 macrophages P65 induced by LPS was significantly inhibited by LA and LA@ZIF-8 (Fig. 6K ). In conclusion, LA and LA@ZIF-8 exert anti-inflammatory effects by inhibiting NF-κB signaling activation, with LA@ZIF-8 demonstrating a superior inhibitory effect.

### 3.7 LA@ZIF-8 biosafety in vivo

**Fig. 7:**
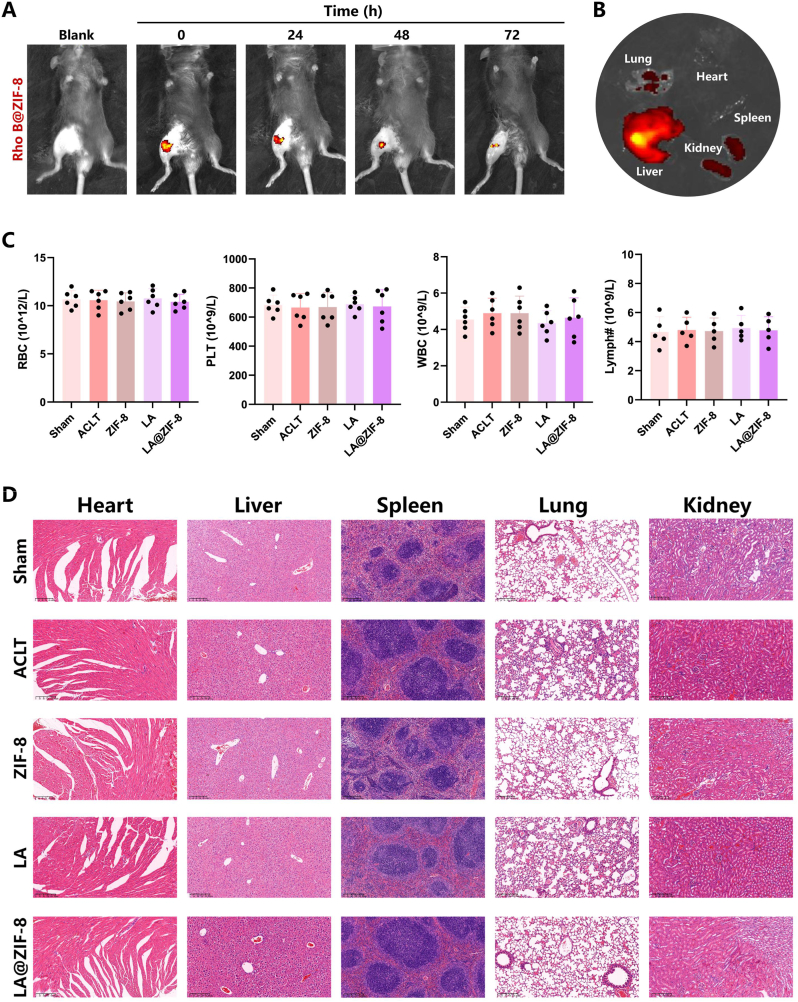
**LA@ZIF-8 biological distribution and histological evaluation on ACLT-induced OA mice** A、B) IVIS images of the biodistribution of LA@ZIF-8-Rho B and its excretion. C) The counts of red blood cells (RBC), platelets(PLT), white blood cells (WBC) and lymphocytes (Lymph#) in whole blood of mice after different treatments. D) Histological analysis of heart, liver, spleen, lung, and kidney in different group by H&E staining. Scale bars are 200 μm. The data are presented as the mean ± SD. ∗P < 0.05, ∗∗P < 0.01, ∗∗∗P < 0.001, n = 6. (For interpretation of the references to colour in this figure legend, the reader is referred to the Web version of this article.)

To determine the biodistribution of LA@ZIF-8, Rhodamine B was encapsulated within LA@ZIF-8 and injected into the articular cavity of an OA mouse model. In vivo, biofluorescence was monitored using an IVIS. The fluorescence signal of Rhodamine B gradually decreased over time until 72 h post-injection (Fig. 7A), suggesting that LA@ZIF-8 was stably retained in the joint cavity for approximately 72 h. Consequently, a follow-up in vivo experiment was conducted by intra-articular injections every three days to maintain the continuous effect of LA@ZIF-8. Additionally, 24 h after the initial injection, LA@ZIF-8 accumulation was observed in the liver, kidney, and other tissues and organs. The strongest fluorescence signal in the liver indicated that it was the main metabolic organ for LA@ZIF-8 (Fig. 7B). To further evaluate the biosafety of LA@ZIF-8, the blood cell counts, including red blood cells, platelets, white blood cells, and lymphocytes, of mice in each group were determined; no significant changes were indicated (Fig. 7C). Additionally, no significant abnormalities were observed in the structure of tissues and organs in any of the groups (Fig. 7D). These results demonstrate that LA@ZIF-8 exhibits excellent biosafety.

### 3.8 LA@ZIF-8 treatment of articular cartilage injury in OA

**Fig. 8:**
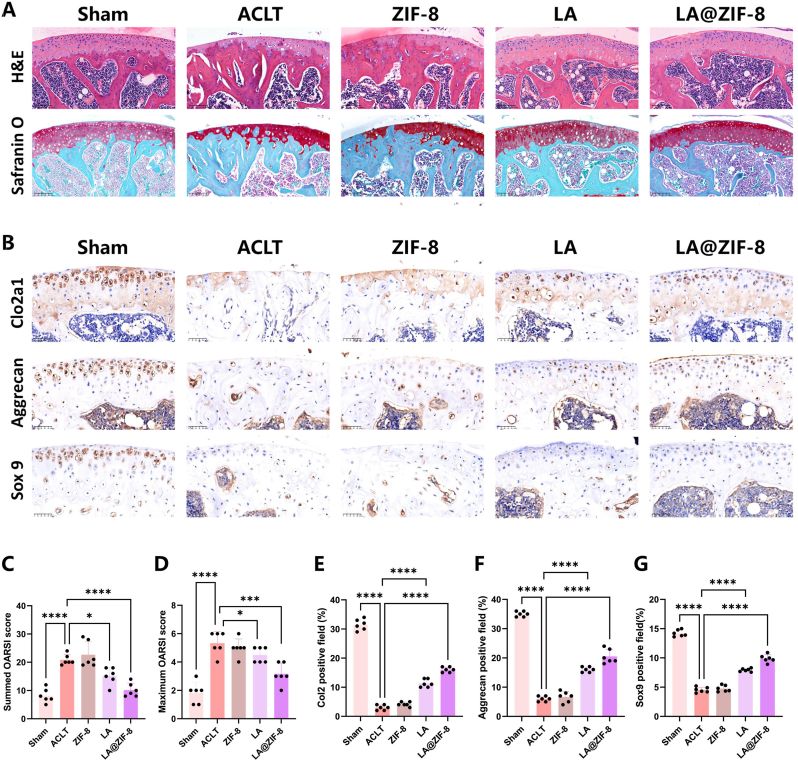
**Effect of LA@ZIF-8 on cartilage degradation of OA model.** A)representative image of H&E and Safranin O. Scale bars are 200 μm. B) IHC staining of Col2a1, Aggrecan and Sox9. Scale bars are 200 μm. C) The maximum OARSI scores was graded to the different groups of cartilage. D) The summed OARSI scores was graded to the different groups of cartilage. E) Quantitative analysis of Col2a1 in the articular cartilage in different group. F) Quantitative analysis of Aggrecan in the articular cartilage in different group. G) Quantitative analysis of Sox9 in the articular cartilage in different group. The data are presented as the mean ± SD. ∗P < 0.05, ∗∗P < 0.01, ∗∗∗P < 0.001, n = 6.

Given the excellent anti-inflammatory and ROS scavenging abilities of LA@ZIF-8 observed in vitro, an OA mouse model was constructed using ACLT to explore its anti-inflammatory effects in vivo (Fig. 8A and B). Compared with the control group, the OA mouse models in each treatment group received different interventions. Severe cartilage wear was observed in the ACLT group, with significantly thinner articular cartilage and active bone reconstruction in the subchondral bone. The damage of articular cartilage in ZIF-8 group was similar to that in ACLT group, but the damage of articular cartilage in the LA group was significantly reduced, and surprisingly, the damage of articular cartilage matrix in the LA@ZIF-8 group returned to a near normal level (Fig. 8A). The Osteoarthritis International Research Society (OARSI) scores were significantly lower (Fig. 8C and D). Expression levels of Col2a1, aggrecan, and SOX-9 increased (Fig. 8E–F-G). In conclusion, LA@ZIF-8 effectively alleviated ACLT-induced articular cartilage injury in vivo and significantly reduced OARSI scores.

### 3.9 LA@ZIF-8 treatment alleviates OA synovitis by inhibiting inflammatory pathways

**Fig. 9:**
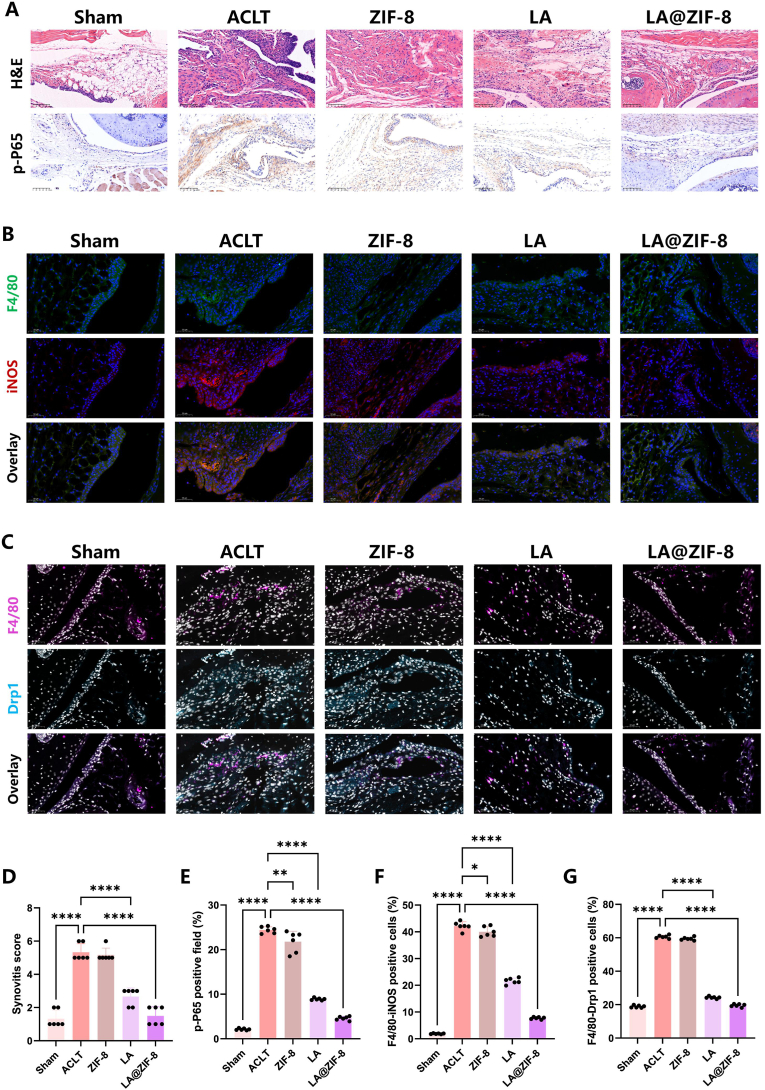
**Effect and mechanism of LA@ZIF-8 on M1 phenotype of synovial macrophages of OA synovitis.** A) Representative images of synovium with H&E and IHC staining of p-P65. B) Immunofluorescence staining of M1-polarization marker (iNOS) and F4/80 in synovial macrophages. C) Immunofluorescence staining of mitochondrial division proteins Drp1 and F4/80 in synovial macrophages. D) The synovitis score of OA mice in each group. E) Analysis of p-P65 positive cell rate in synovial membrane. F) Analysis of F4/80-iNOS positive cell rate in synovial membrane. G) Analysis of F4/80-Drp1 positive cell rate in synovial membrane. The data are presented as the mean ± SD. ∗P < 0.05, ∗∗P < 0.01, ∗∗∗P < 0.001, n = 6.

Joint synovitis cell infiltration, hyperplasia, and synovitis scores in the ACLT and ZIF-8 group were significantly higher than those in the control group, while the synovitis score of mice treated with LA@ZIF-8 was significantly lower (Fig. 9A–D). To verify whether the mechanism by which LA@ZIF-8 delays osteoarthritis is related to a reduced proportion of M1-type macrophages in synovial tissue and inhibition of the NF-κB signaling pathway, we measured the expression levels of p-P65 in each group using immunohistochemical staining. The number of p-P65 positive regions in the synovial tissue of mice in the ACLT and ZIF-8 group was significantly increased, these results indicated that the NF-κB signaling pathway was significantly activated during synovitis, while the expression levels of p-P65 were significantly reduced and the NF-ĸB signaling pathway was inhibited after LA@ZIF-8 treatment (Fig. 9A–E). Furthermore, the macrophage marker F4/80 and M1 polarization marker iNOS were labeled using immunofluorescence. Compared with control mice, the expression of iNOS in the joint synovial tissue of the ACLT group was significantly increased, while the expression of iNOS in the LA and LA@ZIF-8 groups was reduced to varying degrees. Among the groups, LA@ZIF-8 showed the most pronounced inhibition of M1 macrophage polarization (Fig. 9B–F). In a previous in vitro study, LA and LA@ZIF-8 were found to exert therapeutic effects on LPS-induced mitochondrial damage in RAW264.7 macrophages. We also labeled F4/80 and Drp1 using immunofluorescence. The results indicated that F4/80 and Drp1 were co-localised, with significantly increased Drp1 fluorescence expression in the synovium of mice in the ACLT group, while the fluorescence expression of Drp1 in the LA and LA@ZIF-8 groups was significantly reduced after treatment. Additionally, LA@ZIF-8 significantly inhibited Drp1 expression (Fig. 9C–G). In conclusion, LA@ZIF-8 inhibits the activation of NF-κB in the treatment of OA synovitis and suppresses macrophage mitochondrial division, thereby enhancing its anti-inflammatory effect.

## 4 Discussion

**Fig. 10:**
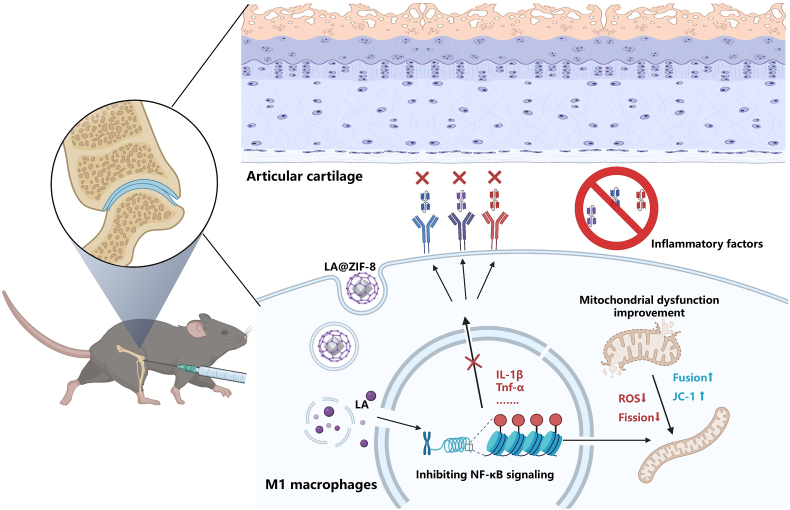
**Schematic diagram of a mechanism to LA@ZIF-8 treat osteoarthritis by inhibiting synovial macrophage-mediated inflammation.** This study found that LA@ZIF-8 inhibited the pro-inflammatory polarization of M1 macrophages, improved mitochondrial function, protected chondrocytes from degradation, and showed good biosafety in vivo with prolonged retention in joint cavities. The findings highlight the potential of LA@ZIF-8 nanoparticles as a novel therapeutic strategy for OA by targeting synovial macrophages, inhibiting inflammatory responses, and protecting cartilage.

This study focused on synovial macrophage inflammation in OA and found that LA targeting OA joint synovitis significantly reduced LPS-induced secretion of M1-type macrophage inflammatory cytokines and protected macrophage mitochondrial function. Transcriptome sequencing showed that LA inhibited LPS-induced NF-κB signaling pathway activation in RAW264.7 cells. To address the limited bioavailability of LA, an LA-etched ZIF-8 nanoparticle was designed and developed. LA@ZIF-8 demonstrated good biosafety, can inhibit the activation of NF-κB in the macrophage inflammatory pathway, and can protect mitochondrial function in vivo and in vitro, ultimately mitigating OA-related cartilage injury. This offers a feasible strategy for the treatment of OA cartilage and provides a theoretical basis for decelerating OA progression (Fig. 10).

The search for effective drugs for early treatment of synovitis to improve the inflammatory microenvironment and prevent its progression has garnered considerable attention. Macrophages derived from the synovium differentiate into at least two subgroups with distinct functions in response to different stimuli. The M1 subgroup is known as classically activated macrophages, which primarily express pro-inflammatory factors and drive inflammation, while the M2 subgroup is known as alternately activated macrophages, which mainly suppress inflammation and contribute to tissue repair. Research has shown that the infiltration of M1 macrophages promotes the differentiation of degenerative and proliferative chondrocytes through multiple mechanisms [9]. OA synovitis is characterised by an increased accumulation of M1 macrophages and ROS in the synovial tissue. Inflammatory synovial macrophages produce cytokines, such as TNF-α, IL-1, IL-6, and iNOS, promoting oxidative stress and degradation of the chondrocyte matrix through paracrine signaling. Therefore, reducing the infiltration of M1-type macrophages into the synovial membrane and reducing the expression of harmful inflammatory factors may represent a potential treatment option for OA [5,6].

Pro-inflammatory factors, such as LPS and IFN-γ, induce the expression of M1-related genes in macrophages, and this metabolic state prevents M1 macrophages from converting to the M2 phenotype because LPS inhibits mitochondrial respiration in macrophages. Both M1 and M2 macrophages expressed CD80 and CD86, with M2 macrophages exhibiting similar inflammatory cytokine secretion and iNOS-mediated nitric oxide production in response to inflammatory factors. M2 macrophages can easily be repolarised into a pro-inflammatory state upon re-stimulation in vitro or in vivo, whereas M1 macrophages are not easily converted into M2 macrophages [34]. Mitochondria are recognised as hubs of signaling pathways that regulate metabolism, redox homeostasis, and cell fate determination. Research indicates that mitochondrial damage activates the classical NF-κB pathway, potentially upregulating the production of inflammatory cytokines. Mitochondrial dysfunction leads to NF-κB pathway activation, with damaged mitochondria recruiting the effector NEMO to activate NF-kB signaling [35]. Mitochondrial dynamics are determined by the balance between division and fusion, the proliferation of mitochondria fission in pro-inflammatory differentiated macrophages, and the interaction between Drp1 and Fis1, which are essential for mitochondrial division [36]. Consequently, the inhibition of macrophage mitochondrial division can suppress the macrophage M1 pro-inflammatory phenotype. Additionally, pro-inflammatory macrophages contain more mitochondria, yet these increased mitochondria exhibit reduced ridge and membrane potentials, often resulting in reduced ATP synthesis and mitochondrial ROS accumulation. This further promotes NF-κB signaling, dependent on inflammatory factor expression [33].

Drug delivery systems have been employed for the diagnosis and treatment of various diseases. Nanoparticles accumulate in damaged or diseased tissues through active or passive targeting mechanisms, releasing drugs in response to microenvironmental factors to achieve therapeutic purposes. Previous studies have shown that the reactive polymer astaxanthin and autophagy activator rapamycin self-assemble into nanoparticles (NP@PolyRHAPM) to inhibit the polarization of M1 macrophages and restore the proliferative capacity of co-cultured chondrocytes [37]. Yang et al. developed PLGA nanoparticles loaded with M1 macrophage cell-masticated viritepofene to enhance M1 macrophage infiltration in the synovium by modulating the YAP1/TXNIP signaling axis, improving OA outcomes [38]. Jin et al. prepared carbon dots (CDs) with good biocompatibility and treated a macrophage-conditioned medium with CDs to reduce IL-1β-induced chondrocyte damage [39]. Among various drug delivery systems, MOFs have gained considerable attention due to their efficient drug delivery capabilities and multimodal imaging properties. MOFs are supramolecular network-structured materials formed by the self-assembly of organic ligands and metal ions. Their key characteristics include a high porosity, efficient drug loading, superior bionic catalytic performance, and excellent biocompatibility [31]. MOFs have been widely applied across various research fields. Among the various MOFs, ZIF-8 containing zinc has garnered significant attention due to its ease of synthesis and superior biocompatibility. A key property of ZIF-8 is its ability to degrade spontaneously in acidic environments, reducing drug release during transport and thereby increasing the concentration of the drug at the lesion site. Moreover, ZIF-8 nanoparticles offer unique advantages in protein loading; they effectively protect internal proteins from external stressors, such as heat, chemical reagents, and mechanical stress, while also providing high drug loading efficiency and rapid drug encapsulation [28]. Through specific chemical interactions, polyphenols coordinate with zinc ions in ZIF-8 to enhance the structural stability of ZIF-8, allowing it to maintain excellent performance under a wider range of environmental conditions. The polyphenol-metal network can not only eliminate the toxic limitations of ZIF-8 in biomedical applications but also reduce resistance across cell membranes, and the resulting nanoparticles can accumulate at positively charged inflammatory sites and undergo controlled degradation in an acidic environment.

## 5 Conclusion

In summary, by using ZIF-8 as the sacrifice template and LA as the etching agent, we designed and developed LA@ZIF-8, Through the coordination of LA and zinc ion coordination, a large amount of LA is attached to the framework. and has good biocompatibility. This system can treat synovial macrophage inflammation and mitochondrial dysfunction through inhibition of the pro-inflammatory NF-κB signaling pathway in OA, thus preventing the degeneration of articular cartilage caused by the inflammatory microenvironment. It is foreseeable that LA@ZIF-8 has clinical potential as a targeted therapy to regulate the microenvironment of synovial inflammation in the early stage and intervene in the progression of OA.

## CRediT authorship contribution statement

**Yu Zhang:** Writing – original draft, Investigation, Formal analysis, Conceptualization. **Qiqi Lou:** Investigation, Formal analysis. **Hao Lian:** Investigation, Formal analysis. **Ran Yang:** Investigation, Formal analysis. **Ruolin Cui:** Methodology, Formal analysis. **Leyang Wang:** Investigation. **Bitao Ma:** Supervision, Methodology. **Lingli Hou:** Supervision, Methodology. **Lilun Jin:** Supervision, Conceptualization. **Weiran Teng:** Writing – review & editing, Supervision, Funding acquisition, Conceptualization.

## Funding

This work was supported by Shanghai "Science and Technology Innovation Action Plan" biomedical science and technology support project (20S21900200).

## Declaration of competing interest

The content is solely the responsibility of the authors. All authors have read and approved the manuscript and have no conflicts of interest to disclose. The funding body was not involved in the design, collection, analysis, and interpretation of data, or the writing of the manuscript.

## Data Availability

Data will be made available on request.
